# Improvement of L-phenylalanine production from glycerol by recombinant *Escherichia coli* strains: The role of extra copies of *glpK, glpX,* and *tktA* genes

**DOI:** 10.1186/s12934-014-0096-1

**Published:** 2014-07-11

**Authors:** Katrin Gottlieb, Christoph Albermann, Georg A Sprenger

**Affiliations:** 1 Institute of Microbiology, University of Stuttgart, Allmandring 31, Stuttgart, 70569, Germany

**Keywords:** Escherichia coli, Glycerol, Crude glycerol, L-phenylalanine, Glycerol kinase, Transketolase, Fructose-1,6-bisphosphatase, Metabolic engineering

## Abstract

**Background:**

For the production of L-phenylalanine (L-Phe), two molecules of phosphoenolpyruvate (PEP) and one molecule erythrose-4-phosphate (E4P) are necessary. PEP stems from glycolysis whereas E4P is formed in the pentose phosphate pathway (PPP). Glucose, commonly used for L-Phe production with recombinant *E. coli,* is taken up via the PEP-dependent phosphotransferase system which delivers glucose-6-phosphate (G6P). G6P enters either glycolysis or the PPP. In contrast, glycerol is phosphorylated by an ATP-dependent glycerol kinase (GlpK) thus saving one PEP. However, two gluconeogenic reactions (fructose-1,6-bisphosphate aldolase, fructose-1,6-bisphosphatase, FBPase) are necessary for growth and provision of E4P. Glycerol has become an important carbon source for biotechnology and reports on production of L-Phe from glycerol are available. However, the influence of FBPase and transketolase reactions on L-Phe production has not been reported.

**Results:**

L-Phe productivity of parent strain FUS4/pF81 (plasmid-encoded genes for *aroF, aroB, aroL, pheA*) was compared on glucose and glycerol as C sources. On glucose, a maximal carbon recovery of 0.19 mM C_Phe_/C_Glucose_ and a maximal space-time-yield (STY) of 0.13 g l^−1^ h^−1^ was found. With glycerol, the maximal carbon recovery was nearly the same (0.18 mM C_Phe_/C_Glycerol_), but the maximal STY was higher (0.21 g l^−1^ h^−1^). We raised the chromosomal gene copy number of the genes *glpK* (encoding glycerol kinase), *tktA* (encoding transketolase), and *glpX* (encoding fructose-1,6-bisphosphatase) individually. Overexpression of *glpK* (or its feedback-resistant variant, *glpK*^G232D^) had little effect on growth rate; L-Phe production was about 30% lower than in FUS4/pF81. Whereas the overexpression of either *glpX* or *tktA* had minor effects on productivity (0.20 mM C_Phe_/C_Glycerol_; 0.25 g l^−1^ h^−1^ and 0.21 mM C_Phe_/C_Glycerol_; 0.23 g l^−1^ h^−1^, respectively), the combination of extra genes of *glpX* and *tktA* together led to an increase in maximal STY of about 80% (0.37 g l^−1^ h^−1^) and a carbon recovery of 0.26 mM C_Phe_/C_Glycerol_.

**Conclusions:**

Enhancing the gene copy numbers for *glpX* and *tktA* increased L-Phe productivity from glycerol without affecting growth rate. Engineering of glycerol metabolism towards L-Phe production in *E. coli* has to balance the pathways of gluconeogenesis, glycolysis, and PPP to improve the supply of the precursors, PEP and E4P.

## Background

**Figure 1:**
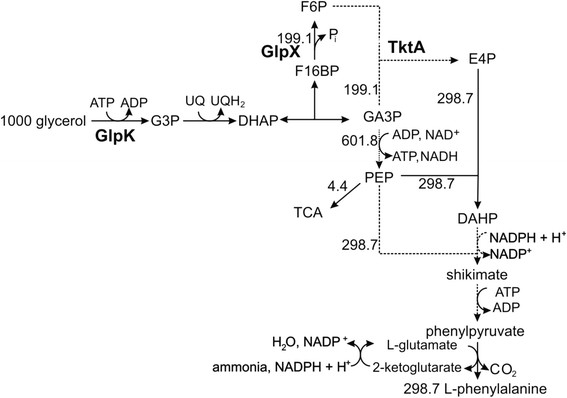
**Pathway and carbon flux from glycerol to L-phenylalanine.** Scheme of carbon flux to L-Phe based on glycerol. Broken arrows indicate an incomplete presentation of the metabolic pathway. The reactions catalyzed by glycerol kinase (GlpK) transketolase (TktA) and fructose-1,6-bisphosphatase (GlpX) are highlighted in bold. Numerics indicate the numbers of molecules from the aforementioned compounds. Based on 1000 molecules of glycerol, 298.7 molecules of L-Phe can be formed.

Glycerol is presently the main carbon byproduct (about 10% w/v) of biodiesel production which is increasing worldwide [[1],[2]]. Glycerol thus is a renewable feedstock which can be utilized as sole carbon- and energy-source by microorganisms like *E. coli.* Under aerobic conditions, the metabolism of glycerol in *E. coli* starts by uptake into the cells either through simple diffusion (relevant if millimolar concentrations are present) or via protein-mediated (GlpF) facilitated diffusion [[3]]. Glycerol is trapped by an ATP-dependent glycerol kinase (GlpK) to yield glycerol-3-phosphate (G3P) [[4]] which is then oxidized by a membrane-bound ubiquinone-8 (UQ_8_)-dependent G3P dehydrogenase (GlpD) to dihydroxyacetone phosphate (DHAP) [[5],[6]] that enters glycolysis (Figure 1). Reduced UQ_8_ passes its electrons along the respiratory chain onto oxygen with the eventual formation of ATP. With glycerol as carbon source, a P/O-ratio of 0.83 has been determined [[7]]. In contrast, glucose, currently the most frequently used carbon and energy source for the microbial production of fine chemicals [[8]], enters the *E. coli* cell mainly through enzymes II of the phosphoenolpyruvate (PEP)-dependent sugar phosphotransferase system (PTS) which transports glucose with concomitant phosphorylation to G6P [[9],[10]]. During metabolism of G6P via the upper Embden-Meyerhof-Parnas pathway, no reducing equivalents are produced until DHAP/glyceraldehyde-3-phosphate (Ga3P) is reached. Catabolic pathways of glycerol and glucose convene at this step and both compounds are metabolized via the lower trunk of glycolysis to PEP and the final product, pyruvate [[11]].

The main drawback for the utilization of glycerol as carbon source for the fermentative production of bulk or fine chemicals is the relatively slow growth of *E. coli* on this carbon source. On glucose, *E. coli* reaches a growth rate (μ) of up to 0.8 h^−1^ [[12]]. In contrast, on glycerol μs are in the range of μ = 0.23 - 0.55 h^−1^ [[11],[13]]. The activity of the glycerol kinase (gene *glpK*) has been described as a limiting factor for the growth on glycerol [[4]]. From the basic understanding of glycerol metabolism, it is known that GlpK is inhibited by fructose-1,6-bisphosphate (F1,6BP) [[14]]. The group of Lin showed that an *E. coli* mutant strain with a GlpK enzyme resistant towards feedback inhibition by F1,6BP displayed a higher growth rate than the wild-type. The doubling time of the mutant on glycerol had decreased from 90 min to 70 min (equivalent to an increase of μ from 0.42 h^−1^ to 0.46 h^−1^) [[4]]. An analysis of glycerol-specific revertants of a *ptsI* mutant of *E. coli* had shown that most revertants had an altered regulation by F1,6BP [[15]].

Furthermore, unphosphorylated molecules of enzyme IIA^Glc^ (EIIA^Glc^) inhibit glycerol kinase and thus provoke inducer exclusion [[9],[16],[17]] as the inducer molecule of the *glp* regulon, G3P, cannot be formed [[14]]. This prevents coutilization of glucose and glycerol and results in diauxic growth [[11]]. Comparisons of *glpK*-mutant strains which were either resistant towards F1,6BP [[15]] or against EIIA^Glc^ [[16]] confirmed that F1,6BP is the main effector of glycerol kinase.

Another limiting step in glycerol metabolism is the necessity for a gluconeogenic formation of G6P, specifically the reaction from F1,6BP to fructose-6-phosphate (F6P) [[18]] catalysed by the enzyme fructose-1,6-bisphosphatase (FBPase). *E. coli* has two isoenzymes (FBPase I and FBPase II) encoded by the genes *fbp* [[19],[20]] and *glpX* [[21]], respectively. Under glycerol-limiting conditions, the flux through the gluconeogenic FBPase is equal to 13% of the glycerol consumption rate [[22]].

Production of valuable compounds e.g. aromatic amino acids such as L-Phe necessitates provision of the building blocks, − PEP and E4P [[23]–[25]]. Whereas PEP formation from glycerol can occur via the lower glycolytic pathway, E4P formation requires functions of the PPP and a connection from gluconeogenesis to the PPP. Thus the enzyme transketolase could play a major role in provision of the precursor E4P [[24]–[27]] when *E. coli* cells grow on glycerol. One of the transketolase reactions is the interconversion of F6P and Ga3P to E4P and xylulose-5-phosphate (X5P) [[26],[28],[29]] and is therefore directly involved in the supply of the L-Phe precursor, E4P.

We show here the utilization of glycerol as carbon and energy source for *E. coli* cells and production of a valuable compound, L-Phe which is a precursor of the artificial sweetener, Aspartame. L-Phe world production exceeds 15.000 tons/year with main production capacities in China [[24],[25],[30]]. The utilization of glycerol as carbon source for the production of aromatic amino acids, and specifically L-Phe, has been shown earlier [[31]–[36]]. To our best knowledge, however, no attempts have been made to improve aromatic amino acid production, and especially L-Phe, from glycerol as sole carbon source by improving gluconeogenic and PPP reactions. We also show that crude glycerol, the byproduct of biodiesel production can be used for L-Phe production.

## Results

### Production strain *E. coli* FUS4/pF81

We intended to study L-Phe production from glycerol as sole carbon and energy source. As a model for strain development, we used derivatives of *E. coli* K-12 wild type strain W3110 (laboratory name LJ110, [[37]]). A double auxotroph mutant (F4, auxotrophic for L-Phe and L-tyrosine) was engineered by deletion of the chromosomal genes *pheA, aroF*, and *tyrA* [[38]] which are adjacent on the *E. coli* chromosome. To convert this strain into an L-Phe producer, it had been transformed with plasmid pF81 (Amp^R^; *lacI*^q^/P*tac*-dependent promoter) which carries an artificial operon consisting of the genes *aroF*, *pheA’*, *aroB*, and *aroL* (see Table 1 in Materials and Methods) [[38]]. This gene combination had been shown earlier to result in a L-Phe producer which had high L-Phe productivity in shaking flasks or fermenters while low acetic acid or other by-products were formed with glucose as sole C- and energy source [[25],[38]–[40]]. It has to be noted that plasmid pF81 carries the genes *aroF* (for the tyrosine-sensitive DAHP-Synthase AroF), and *pheA’* (encoding a C-terminally shortened chorismate mutase/prephenate dehydratase PheA) which renders PheA resistant towards the final product, L-Phe. Strain F4/pF81 remains auxotrophic for L-tyrosine; this feature can be used to control biomass formation [[38],[40]]. Due to the notorious plasmid loss during cultivations (even with intermittent addition of ampicillin) we decided to enhance the activities of AroF, AroB, and AroL further. Therefore, an artificial operon of the three genes (*aroF*, *aroB*, and *aroL* under a P*tac*-promoter control) was chromosomally inserted into the *lac* gene locus [[41],[42]]. The resulting strain, FUS4 (see Table 1; Materials and Methods), was the parent strain for further genetic manipulations described in this work and others [[31],[43]].

**Table 1:** Strains and plasmids used in this study

**Strain or plasmid**	**Relevant Genotype all strains are** ***E. coli*** **K-12**	**Reference**
LJ110	Wildtype W3110 (F^−^, λ^−^, IN (*rrnD-rrnE*) 1, *rph*-1)	[[37],[44]]
BW25113	*lac*I^q^*rrnB*_T14_ ∆*lacZWJ*_WJ16_*hsd*R514	[[45]]
	∆*ara*BAD_AH33_ ∆*rha*BAD_LD78_	
XL1-Blue	*recA1 endA1 gyrA96 thi-1 hsdR17 supE44 relA1 lac[F’proABlacIqZ*Δ*M15Tn10(Tet*^*r*^*)]*	(Stratagene, La Jolla, USA)
F4	LJ 110 Δ (*pheA tyrA aroF*)	[[38]]
F144	LJ 110 Δ*lac*::P_tac_::*aroFBL*	J.Bongaerts, unpubl. [[42]]
FUS4	LJ110 Δ (*pheA tyrA aroF*) Δ*lac*::P_tac_::*aroFBL*^*+*^ *=* F4 x P1-lysate F144	G. A. Sprenger and N. Trachtmann, unpubl.
BW25113 Δ*tktA*	BW25113 Ara^+^ Δ*tktA*	N. Trachtmann and G.A. Sprenger, [[42]]
BW25113 Δ*tktA tktA*^*+*^	BW25113 Ara^+^ Δ*tktA gal::*P_tac_*::tktA-cat*	N. Trachtmann, [[42]]
BW25113 *glpK*^+^	BW25113 Δ*mal::*P_tac_*::glpK-cat*	This work
BW25113 *glpK*^G232D^	BW25113 Δ*mal::*P_tac_*::glpK* G232D*-cat*	This work
LJ110 Δ*glpK*	LJ110 Δ*glpK-km*	This work
LJ110 Δ*glpK glpK*^+^	LJ110 Δ*glpK-km* Δ*mal::*P_tac_*::glpK-cat =* LJ110 Δ*glpK* x P1-lysate BW25113 *glpK*^+^	This work
LJ110 Δ*glpK glpK*^G232D+^	LJ110 Δ*glpK-km* Δ*mal::*P_tac_*::glpK*G232D*-cat =* LJ110 Δ*glpK* x P1-lysate BW25113 *glpK*^G232D+^	This work
BW25113 *glpX*^+^	BW25113 Δ*rbs::*P_tac_*::glpX-cat*	This work
FUS4.2	FUS4 Δ*mal::*P_tac_*::glpK-cat =* FUS4 x P1-lysate BW25113 *glpK*^+^	This work
FUS4.2-1	FUS4 Δ*mal::*P_tac_*::glpKG232D-cat =* FUS4 x P1-lysate BW25113 *glpK*^*fbr+*^	This work
FUS4.5	FUS4 Δ*gal::*P_tac_*::tktA-cat =* FUS4 x P1-lysate BW25113 Δ*tktA tktA*^*+*^	This work
FUS4.6	FUS4 Δ*rbs::*P_tac_*::glpX-cat =* FUS4 x P1-lysate BW25113 *glpX*^+^	This work
FUS4.6-2	FUS4 Δ*rbs::*P_tac_*::glpX*	This work
FUS4.7	FUS4 Δ*rbs::*P_tac_*::glpX* Δ*gal::*P_tac_*::tktA-cat =* FUS4.6-2 x P1-lysate BW25113 Δ*tktA tktA*^*+*^	This work
pKD46	λ Red disruption system (γ, β, exo under control of *araB*p), Amp^R^	[[45]]
pCP20	*FLP*^*+*^*, λ* ci857^+^, *λ ρ*_R_ Rep^ts^, Amp^R^, Cm^R^	[[46]]
pCO1kanFRT	Amp^r^ Km^r;^ Kanamycin resistance gene flanked by FRT sites	U. Degner and G.A. Sprenger, unpubl. [[42]]
pCO1catFRT	Ap^r^ Cm^r;^ chloramphenicol resistance gene flanked by FRT sites	U. Degner and G.A. Sprenger, [[42]]
pJF119EH	P_tac_, RBS, Amp^R^, *lac*I	[[47]]
pF81	P_tac_::*aroF, pheA*^*fbr*^*, aroB, aroL* Amp^R^, *lac*I	[[25],[38]]b
pKUS3	1,5 kb *glpK*-PCR-Product (*Nde* I → blunt/*Hind* III) from *E. coli* LJ110 (W3110) in pJF119EH (*EcoRI* → *blunt*/*Hind*III)	This work
pKUS3-1	pKUS3 with Quikchange *glpK* G695A (bp)	This work, according to [[48]]
pKUS10	pKUS3 with a chloramphenicol-resistance cassette in *Sph* I-site	This work
pKUS13	1,5 kb *glpX*-PCR-Product (*Nde* I → blunt/*Hind* III) from *E. coli* LJ110 (W3110) in pJF119EH (*EcoRI* → *blunt*/*Hind*III)	This work
pKUS14	pKUS13 with a chloramphenicol-resistance cassette in *Sph* I-site	This work

### Effect of carbon source (glucose, glycerol, crude glycerol) and pO_2_ on L-phenylalanine productivity in fed-batch cultivations

**Figure 2:**
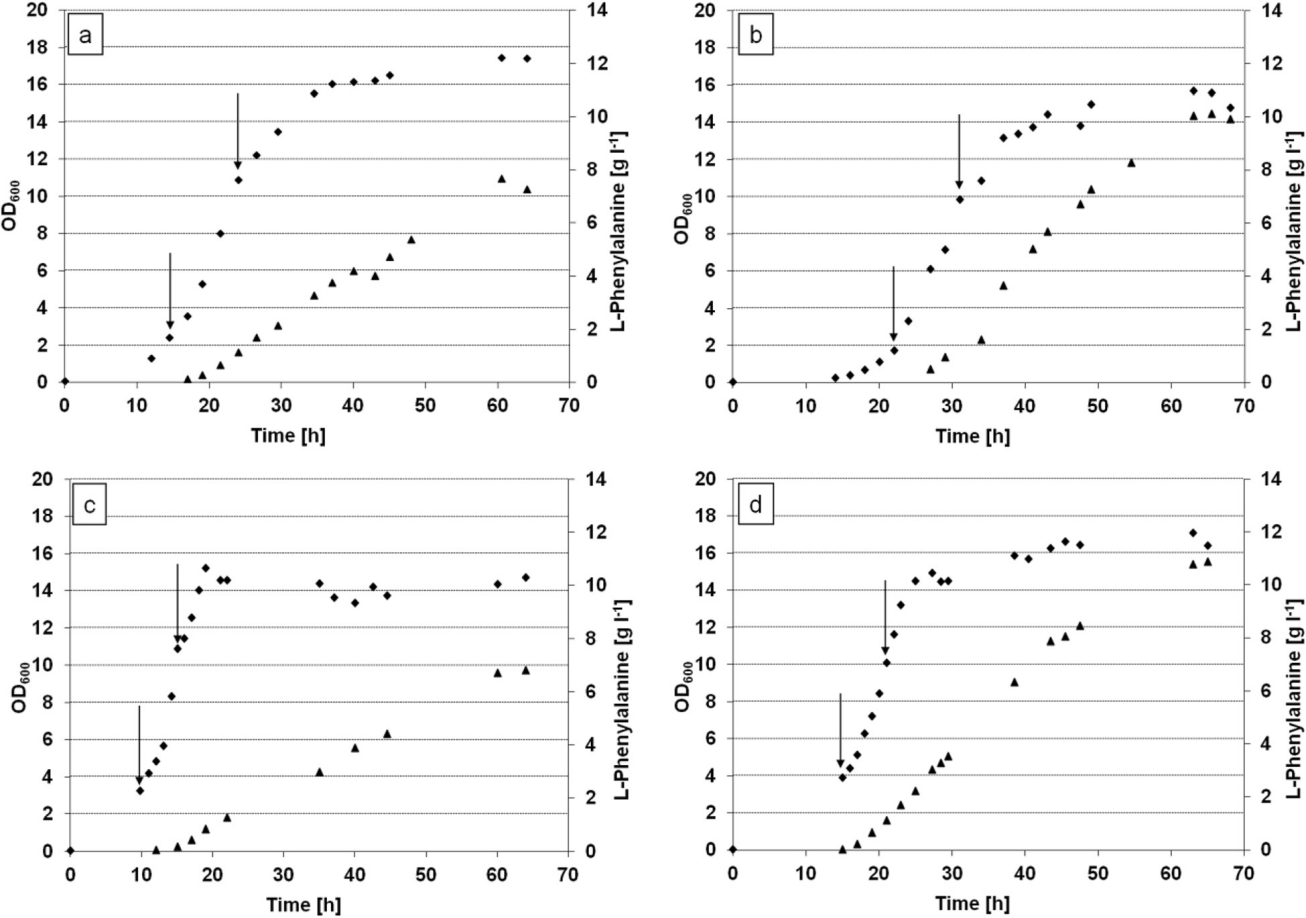
**In order to compare (glucose, glycerol, crude glycerol) and pO**_**2**_**on L-phenylalanine productivity in fed-batch cultivations.** Strain FUS4/pF81 was cultivated in fed-batch mode under different conditions. **(a)** with glycerol at 60%, **(b)** or 20% O_2_ saturation, **(c)** with glucose (60% O_2_), or **(d)** with crude glycerol (20% O_2_) as carbon source. The arrows indicate the time point when IPTG was added for induction. Data for growth rate, carbon yield, space-time-yield, and absolute concentration of L-Phe are listed in Table 2. All fermentations were conducted in MM as described in Materials and methods and were performed two times independently. The mean values are given in Table 2. Diamonds indicate OD_600_ values, triangles indicate L-Phe concentration [g l^−1^].

In order to compare the L-Phe productivity of *E. coli* FUS4/pF81, fed-batch experiments were performed on glucose or glycerol as carbon and energy sources (Figure 2a-d). For glycerol, both pure glycerol (86%) as well as crude glycerol (83.2%) was used. Cells were cultivated in a 3.6 l-bioreactor at 37°C as described in Materials and Methods. As can be seen from Figure 2 and Table 2, growth rate was higher with glucose, reaching μ = 0.49 ± 0.01 h^−1^ whereas on glycerol, growth rate was μ = 0.27 ± 0.01 h^−1^. However, L-Phe productivity was higher with glycerol reaching a space-time-yield (STY) of 0.21 ± 0.02 g l^−1^ h^−1^ and a carbon recovery of 0.18 ± 0.02 mM C_Phe_/C_Gly_. In comparison, STY under our conditions with glucose was 0.13 ± 0.01 g l^−1^ h^−1^ with a similar carbon recovery (0.19 ± 0.02 mM C_Phe_/C_Glc_). Crude glycerol from biodiesel could also be utilized by *E. coli* cells for the production of L-Phe. The STY with crude glycerol (at 20% oxygen partial pressure) was 0.22 ± 0.05 g l^−1^ h^−1^ and the carbon recovery reached 0.23 ± 0.05 mM C_Phe_/C_Gly_. We take this as evidence that crude glycerol can substitute for pure glycerol. This had recently also been shown for another strain that produced L-Phe [[35]]. However, as we noticed rather high standard deviations with crude glycerol preparations (possibly due to impurities in the crude glycerol) we decided to continue our subsequent studies with pure glycerol as carbon and energy source.

**Table 2:** Comparison of the results from the fed-batch fermentations with FUS4/pF81 and its derivatives

**Strain**	**Growth rate**	**Maximal carbon recovery Y C**_**Phe**_**/C**_**Gly**_	**Maximal space-time-yield**	**Maximal concentration L-Phe**	**Lactate conc.**	**Acetate conc.**
	(μ [h^−1^])	[mM] ([%])	[g l^−1^ h^−1^] ([%])	[g l^−1^] ([%])	[mM]	[mM]
FUS4/pF81	0.27 ± 0.01	0.18 ± 0.02 (100)	0.21 ± 0.02 (100)	7.9 ± 0.2 (100)	30.7 ± 10.5	9.5 ± 5.5
FUS4/pF81 (20% O_2_)	0.26 ± 0.01	0.26 ± 0.03 (144.4)	0.26 ± 0.01 (123,8)	9.4 ± 0.7 (119,0)	n.d.*	n.d.
FUS4/pF81 (Glucose)	0.49 ± 0.01	0.19 ± 0.02 (105.6)	0.13 ± 0.01 (61.9)	5.5 ± 1.3 (69.6)	15.4 ± 4.7	3.0 ± 0.7
FUS4/pF81 (crude glycerol, 20% O_2_)	0.32 ± 0.01	0.23 ± 0.05 (127.8)	0.22 ± 0.05 (104.8)	10.0 ± 0.9 (126.6)	46.8 ± 1.5	24.9 ± 6.8
FUS4.5/pF81	0.28 ± 0.02	0.21 ± 0.04 (116.7)	0.23 ± 0.01 (109.5)	9.7 ± 0.8 (124.0)	69.1 ± 9.5	21.9 ± 3.7
FUS4.6/pF81	0.29 ± 0.03	0.20 ± 0.01 (111.1)	0.25 ± 0.02 (119.0)	7.3 ± 0.5 (92.4)	21.1 ± 1.4	3.2 ± 0.4
FUS4.7/pF81	0.24 ± 0.01	0.26 ± 0.06 (144.4)	0.37 ± 0.01 (180.5)	10.1 ± 0.2 (127.8)	31.3 ± 5.0	7.9 ± 3.8

n.d. not detectable, *lactate was not detectable as long as the glycerol concentration stayed below 5 g/l.

All fed-batch fermentations were conducted two times independently in MM in a 3.6 l bioreactor (as described in Materials and methods). If not stated otherwise, (in brackets) pure glycerol was used as carbon source and oxygen saturation was at 60%. The values represent the mean value and the standard deviation.

Acetate and lactate had accumulated as byproducts during both, glucose and glycerol fermentations and also with crude glycerol. With glycerol, up to 40 mM lactate and 15 mM acetate were produced whereas with glucose the concentrations reached 20 mM lactate and 3.0 mM acetate. With crude glycerol, ca. 25 mM acetate and 50 mM lactate were found.

We observed further that a decrease in the oxygen partial pressure (pO_2_) from 60% to 20% during the cultivation on glycerol was followed by an increase in STY (0.26 ± 0.01 g l^−1^ h^−1^) and carbon recovery (0.26 ± 0.03 mM C_Phe_/C_Gly_); in contrast, less glycerol was consumed during a fed-batch regime at low oxygen tension (from 124.7 ± 10.8 g to 99.4 ± 4.3 g). No byproducts were seen when the glycerol concentration stayed below 5 g l^−1^during the cultivation with low pO_2_. In the absence of an exhaust gas analysis, however, we were not able to investigate this finding in more depth.

Since our aim was to combine a high glycerol conversion with an optimal carbon distribution and a high L-Phe production, all following fermentations were performed at 60% oxygen availability where we had measured the highest glycerol consumption.

### Influence of additional chromosomal copies of *glpK* alleles on L-Phe production

Having shown that glycerol is a favourable carbon source for L-Phe production with *E. coli*, we investigated whether genetic alterations of glycerol metabolism might even further improve productivity. It had been described that in wildtype *E. coli* cells, the catabolism of glycerol is limited by the activity of glycerol kinase [[4]]. Furthermore, a point mutation at position 232 (Gly → Asp exchange, G232D) of the protein had partially relieved the enzyme from feedback inhibition by F1,6BP [[48],[49]]. We therefore tried to enhance growth rate and L-Phe production in FUS4/pF81 by the chromosomal integration of an additional gene copy of either *glpK* or of *glpK*^*G232D*^. We therefore cloned the wild type *glpK* gene first on a plasmid (pKUS3, Table 1) and then reconstituted the point mutation (G695A in the gene sequence leading to a G232D exchange in the amino acid sequence) by site-directed mutagenesis (plasmid pKUS3-1, Table 1). Glycerol kinase activity was measured using the crude extract as described in Material and Methods. We could confirm that feedback inhibition by F1,6BP was exerted upon the wild type glycerol kinase as its activity was reduced to 20% in the presence of 10 mM F1,6BP. In contrast, the G232D enzyme variant retained 70% of its activity in the presence of 10 mM of the effector (Table 3).

**Table 3:** Enzyme activities of fructose-1,6-bisphosphatase II and glycerol kinase in the crude extract

**Strain**	**FBPase activity [U mg crude extract**^**−1**^**]**	**Glycerol kinase activity [U mg crude extract**^**−1**^**]**	
		0 mM FBP	10 mM FBP
FUS4	0.53 ± 0.21	-	-
FUS4.6	0.97 ± 0.24	-	-
DH5α pJF119	0.42 ± 0.22	1.5 ± 0.3	0.2 ± 0.1
DH5α pKUS13	3.05 ± 0.62	-	-
DH5α pKUS3	-	52.7 ± 4.1	11.2 ± 0.9
DH5α pKUS3-1	-	64.4 ± 3.6	43.3 ± 3.8

Cells were grown and induced with IPTG as described in Materials and methods. FBPase and glycerol kinase activity was measured in cell-free extracts. DH5α/pKUS13 (carrying gene *glpX*) was compared to the control, DH5α/pJF119EH (empty vector). DH5α/pKUS3 (carrying gene *glpK*) and DH5α/pKUS3-1 (carrying gene *glpK*^*G232D*^) were also compared to the control, DH5α/pJF119EH. After chromosomal integration of *glpX* FBPase activity was measured in the cell-free extract of the resulting strain FUS4.6 and as control in the parent strain, FUS4. FBPase activity was measured by quantifying phosphate release using a malachite green/ammonium molybdate solution and glycerol kinase activity was measured using a pyruvate kinase and lactate dehydrogenase coupled assay as described in Materials and methods [[50]].

The values represent the mean value and the standard deviation.

Next, to check the functional activity of both alleles *in vivo*, we integrated either *glpK* or *glpK*^*G232D*^ in mutant strain LJ110 ∆*glpK* which had been made unable to metabolize glycerol due to a *glpK* knockout (see Table 1). Both genes were integrated into the *mal* gene locus (under the control of a P*tac*-promoter) following described methods [[45],[51]–[53]]. On MacConkey agar pH indicator plates with 1% glycerol, it became visible that each of the *glpK* alleles was able to reconstitute a glycerol-positive phenotype (acid formation from glycerol) of LJ110 ∆*glpK*.

Then we introduced the *glpK* alleles into FUS4 strain (which has one copy of wild type *glpK*) to come up with strains FUS4.2 (additional chromosomal gene copy of *glpK*) and FUS4.2-1 with a gene copy of the feedback resistant *glpK*^*G232D*^. After transformation with the plasmid pF81, we cultivated the strains FUS4.2/pF81 and FUS4.2-1/pF81 in shaking flasks with glycerol as sole carbon source in order to compare their growth behaviour and L-Phe production with the parent strain, FUS4/pF81.

**Figure 3:**
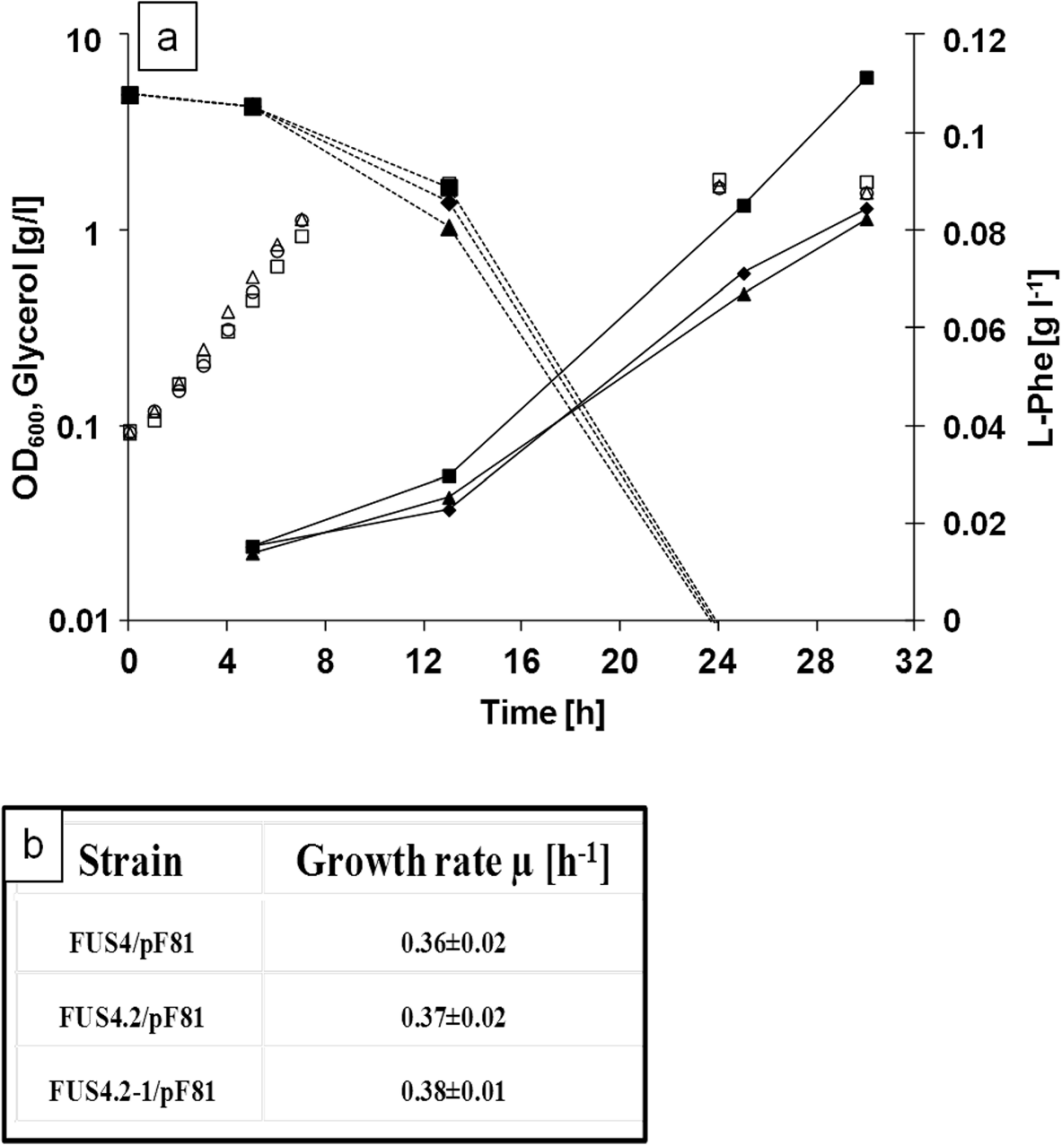
**Effect of additional copies of*glpK*or*glpK***^***G232D***^**on batch growth and L-phenylalanine production.** Strains were grown in MM + 0.5% glycerol in batch cultures as described in Materials and methods. Cultures were analyzed regarding **(a)** cell growth of FUS4/pF81 (open squares), FUS4.2/pF81 (open circles, additional chromosomal copy of *glpK*), and FUS4.2-1/pF81 (open triangles, additional chromosomal copy of *glpK*^*G232D*^), and L-Phe production in [g l^−1^] of FUS4/pF81 (filled squares, full line), FUS4.2/pF81(closed circles, full line), and FUS4.2-1/pF81 (filled triangles, full line). The dotted line shows glycerol consumption of FUS4/pF81 (filled squares), FUS4.2/pF81 (closed diamonds), and FUS4.2-1/pF81 (filled triangles). **(b)** Growth rates were determined during the time period of 1–7 h in **(a)**.

We found that none of the added *glpK* alleles had a significant influence on growth behaviour (Figure 3). The growth rates were slightly increased compared to the bioreactor. For the time period from 1–7 hours, growth rates were μ = 0.36 ± 0.02 h^−1^ for FUS4/pF81, μ = 0.37 ± 0.02 h^−1^ for FUS4.2/pF81, and 0.38 ± 0.01 h^−1^ for FUS4.2-1/pF81, respectively. Since the strain FUS4/pF81 had shown a growth rate of μ = 0.49 ± 0.01 h^−1^ in a medium with glucose as carbon source, we can exclude the possibility that growth was limited by components of the mineral medium other than the C source. Compared to strain FUS4/pF81 which (after 30 h of incubation) had produced 0.11 g l^−1^ of L-Phe- both FUS4.2/pF81 and FUS4.2-1/pF81 yielded about 0.08 g l^−1^ of L-Phe (Figure 3). As can be seen in Figure 3, glycerol consumption was slightly increased in FUS4.2/pF81 and FUS4.2-1/pF81 and lowest in FUS4/pF81. Therefore we concluded that extra copies of either *glpK* or *glpK*^fbr^ did not improve L-Phe production from glycerol.

### Integration of an extra gene copy of *glpX* in *E. coli* FUS4

We looked at further possible limitations of glycerol catabolism. Since growth on glycerol as sole C source requires gluconeogenesis, the enzyme FBPase could become limiting [[18]]. *E. coli* has two *bona fide* FBPase enzymes, encoded by genes *fbpA* and *glpX* [[19]–[21]], the latter is encoded in an operon together with *glpF* and *glpK* [[54]] and is expressed together with these genes [[55]]. We decided to work with GlpX in the present study.

The *glpX* gene was first cloned into the expression vector pJF119EH [[47]] to yield plasmid pKUS13; FBPase enzyme activity was determined in pKUS13-recombinant strains. As can be seen from Table 3, we found a fivefold higher enzyme activity (measured as phosphate release from F1,6BP) in cell-free extracts from strain DH5α/pKUS13, compared to those from a control strain (DH5α/pJF119EH) which proved that the gene *glpX* encoded a functional FBPase activity. Next, the gene *glpX* was integrated into the *rib* locus of *E. coli* FUS4 (for details see Table 1 and Materials and Methods section). We confirmed the proper integration of the gene by incubation of the strain on MacConkey agar plates (supplemented with 10 g l^−1^ of ribose) which gave a pale colony phenotype compared to red colonies of control strains (data not shown). With one added copy of *glpX* on the chromosome, the FBPase enzyme activity in the new strain, FUS4.6, was nearly doubled in contrast to the parent strain (Table 3).

### Growth and L-phenylalanine production with an additional chromosomal copy of *glpX*

**Figure 4:**
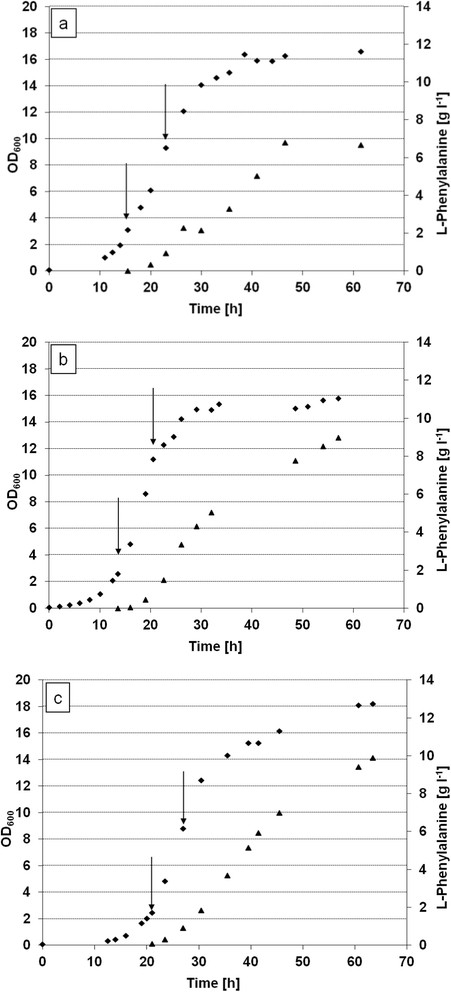
**Fed-batch cultivations of FUS4/pF81 mutants with putatively increased flux through the PPP.** Fed-batch fermentations were conducted in MM with pure glycerol as carbon source. Cell growth (diamonds) and L-Phe concentrations [g l^−1^] (squares) are shown in the diagrams a-c. The arrows indicate induction with IPTG. Data for carbon yield, space-time-yield and absolute concentration of L-Phe are listed in Table 2. All fermentations were performed two times independently, one typical growth curve is presented. **(a)** FUS4.6/pF81 (additional gene copy of *glpX*), **(b)** FUS4.5/pF81 (additional gene copy of *tktA*), **(c)** FUS4.7/pF81 (additional gene copy of *tktA* and *glpX*).

Strain FUS4.6 was transformed with plasmid pF81. The growth rate of strain FUS4.6/pF81 in the bioreactor was nearly unaltered (μ = 0.29 ± 0.03 h^−1^ compared to μ = 0.27 ± 0.01 h^−1^ of FUS4/pF81). Production of L-Phe was slightly improved with a STY of 0.25 ± 0.02 g l^−1^ h^−1^ and a carbon recovery of 0.20 ± 0.01 mM C_Phe_/C_Gly_ (Figure 4a and Table 2). The analysis of the byproducts revealed that the amounts of lactate and acetate were decreased to 20 mM and 3 mM, respectively. This is about half of the byproduct level of the parent strain, FUS4/pF81 (cultivated at 60% oxygen availability) found under these conditions. However, FUS4.6/pF81 showed less glycerol consumption (ca. 90 g during 60 hours), compared to about 124 g in the parent strain.

### Construction of a double knock-in mutant of *E. coli* with additional chromosomal gene copies of *glpX* and *tktA*

Transketolase is an important enzyme of *E. coli* PPP [[28]]. Among other features, loss of *tktA* gene in *E. coli* results in impaired growth on LB agar plates which are supplemented with bile acids [[56]] or with sodium dodecyl sulfate (SDS) (0.25%). The increased sensitivity to SDS is due to a lack of sedoheptulose 7-phosphate formation by the transketolase. This in turn leads to a lack of heptose moieties in the lipopolysaccharide layer of the outer membrane [[57]]. Strain *E. coli* BW 25113 Ara^+^ Δ*tktA* (N Trachtmann & GA Sprenger, unpubl. results) is deleted for transketolase gene *tktA* (see Table 1) and showed increased sensitivity towards 0.25% SDS (see Additional file 1). This strain was used as first recipient for the chromosomal integration of *P*_*tac*_*::tktA-cat* gene cassette. The cassette was inserted into the *gal* locus to yield strain *BW25113 ΔtktA gal::P*_*tac*_*::tktA-cat.* Growth and colony formation on LB agar plates with 0.25% SDS was fully restored in this strain (see Additional file 1). We took these results as evidence that a functional gene copy of *tktA* had been chromosomally integrated. Using phage P1-mediated transduction [[52]], the *gal*::*P*_*tac*_*::tktA-cat* cassette was then introduced into strains FUS4 and FUS4.6.2 (descendant from FUS4.6 without cat-cassette, see Table 1) with selection for chloramphenicol resistance. Strain FUS4 with an extra copy of *tktA* was termed FUS4.5, and with extra copies of both genes, *glpX* and *tktA,* was termed FUS4.7 (Table 1).

### Phenylalanine production in *E. coli* with an additional gene copy of *tktA,* or a combination of *glpX* and *tktA*

Both strains, FUS4.5 and FUS4.7, were then transformed with plasmid pF81 and analyzed for their phenylalanine production in bioreactors. Cultivation and analysis of strain FUS4.5/pF81 showed that an extra *tktA* gene copy had only little effect on growth behaviour and L-Phe production. Growth rate on glycerol was μ = 0.28 ± 0.02 h^−1^, STY for L-Phe was 0.23 ± 0.01 g l^−1^ h^−1^, and carbon recovery was 0.21 ± 0.04 mM C_Phe_/C_Gly_ (Figure 4b, Table 2). The glycerol consumption reached 135 g during 60 hours and was thus slightly higher than in the parent strain, FUS4. The analysis of the byproducts, however, showed an increase in lactate and acetate production, reaching up to 80 mM and 26 mM of these compounds, respectively. In comparison, strain FUS4.7/pF81 with extra gene copies of *glpX* and *tktA* showed a slightly decreased growth rate of μ = 0.24 ± 0.01 h^−1^, but glycerol consumption was about equal compared to the strain FUS4/pF81. L-Phe production was increased to a STY of 0.37 ± 0.01 g l^−1^ h^−1^ and a carbon recovery of 0.26 ± 0.06 mM C_Phe_/C_Gly_ (Figure 4c, Table 2). Compared to the parent strain cultivated under the same conditions, this is an increase of 44.4% regarding carbon recovery and an increase of 80.5% regarding STY.

## Discussion

Glucose is generally the preferred single carbon source for growth and product formation with *E. coli* cells [[9],[12],[58]]. However, glycerol which has become a readily available and unexpensive carbon source as a byproduct of biodiesel production [[1],[2]] also shows considerable advantages. Here, to allow a comparison of molecules with different carbon lengths (glucose with 6C atoms equal to two C3 units, glycerol with 3 C atoms, equal to one C3 unit), three carbon units (C3) shall be regarded. Under aerobic conditions, *E. coli* cells gain less ATP via their respiratory chain (oxidative phosphorylation) from glucose C3 units due to the lower grade of reduction [[7],[59]]. Therefore, glycerol -which is a more reduced C3 unit could yield higher amounts of biomass and products, especially when compounds with a higher grade of reduction, or chemicals which afford high ATP-input are regarded. Figure 1 outlines the major ATP- and NADPH-consuming steps in formation of L-Phe. For example, for the regeneration of L-glutamate as amino donor, one molecule NADPH + H^+^ is necessary [[60]]. Glycerol with its higher reduction potential could ultimately lead to improved NADPH + H^+^ supply, and thus could be of advantage here when compared to glucose.

Thus, several reports describe the fermentative production of fine chemicals based on glycerol with recombinant *E. coli* cells [[2],[31],[33],[61]]. As glycerol metabolism does not need stoichiometrical amounts of phosphoenolpyruvate (PEP) for its uptake (but the glucose phosphotransferase system (PTS) does [[9]]), it can be expected that especially the formation of compounds which utilize the building block PEP (e.g. intermediates from the aromatic amino acid pathway as shikimic acid or final products as L-Phe) should be favored [[62],[63]]. Indeed, at the example of shikimate production, an intermediate of the aromatic amino acid pathway, it had been shown that the theoretical and experimental carbon recovery is highest with glycerol, whereas the space-time-yield was higher with glucose [[62]]. Also for the production of L-Phe, a higher production capability was calculated when using glycerol (0.6 mol/6 C atoms) instead of glucose (0.529 mol/6 C atoms) [[32]]. L-Phe formation requires two molecules of PEP and one erythrose 4-phosphate (E4P) (see Figure 1) [[23]]. L-Phe production with *E. coli* strains usually is based on glucose as carbon source [[10],[24]–[26],[36],[38]–[40]]. For a recent example, strain F4/pF81, the precursor strain of FUS4/pF81 that was used in our investigations, had shown a carbon recovery of 20-26%. A STY of 1.05 g l^−1^ h^−1^ had been reached under pH-, glucose and L-tyrosine control in a fed-batch mode (final OD_600_ in the range between 40 and 50 [[25],[38]]. L-Phe production based on glycerol has recently been described by several groups with single glycerol [[31],[33]–[35],[41],[43]]. Srinophakun and co-workers reported a final L-Phe concentration of nearly 35 mg l^−1^ using an *Escherichia coli* BL21(DE3) strain which expressed a recombinant gene for a L-phenylalanine dehydrogenase from *Acinetobacter lwoffii* [[35]]. The group of Bolivar & Gosset recently showed that even in a wildtype *E. coli* strain the utilization of glycerol yielded traces of aromatic compounds whereas the utilization of glucose did not result in the production of aromatic compounds [[11]]. So obviously glycerol can be considered as a favourable carbon source for the production of aromatic compounds including L-Phe.

Since the model strain FUS4/pF81 had relatively slow growth on glycerol, we decided to work with lower cell densities than described in the work of Rüffer et al. [[38]]. This allowed better reproducibility and easier handling. However, a direct comparisons to the optimized control systems (including fine-tuned L-tyrosine dosage) in fed-batch bioreactors as shown previously with similar *E. coli* strains [[38]] cannot be done here. Working with the L-Phe producing strain FUS4/pF81 as model producer organism, we show here, that in our system the carbon recovery from glucose was in about the same range as described earlier [[38]]. The STY, however, was clearly lower reaching about 0.13 ± 0.01 g l^−1^ h^−1^(Table 2). This can be at least partially attributed to the lower cell density used in our investigation.

We could show that in our system with the carbon sources pure glycerol or crude glycerol, L-Phe production resulted in a higher carbon recovery than using glucose (Table 2). Also the STY was significantly higher.

Strain improvements leading to increased L-Phe titers in *E. coli*, up to now has mainly done considering the supply of building blocks as PEP and E4P, and to overcome existing regulations of the aromatic amino acid pathway [[10],[24]–[27],[36],[38],[64]]. While this has led to impressive improvements of L-Phe production from glucose, less attention has been paid to the pathway engineering for glycerol. Very recently, knock-out strains based on the same chassis strain, FUS4, which lack the genes for both pyruvate kinases (*pykA, pykF*) have been described [[31]]. As glycerol catabolism is almost completely blocked in those *pyk*-negative strains, PEP provision is augmented which can be used to provide more building blocks for L-Phe synthesis. For biomass formation, however, this requires the feeding of a gluconeogenic C source such as lactate [[31]]. The productivity of this strain yielded 22 mg_L-phe_ g_CDW_^−1^ h^−1^. Deletions of the genes *maeA* (encoding NAD-dependent malic enzyme) and *maeB* (encoding NADP-dependent malic enzyme) have not led to improvements in L-Phe production [[43]].

Specific steps in the metabolism of glycerol for product formation have been less discussed. As it had been reported that the activity of the glycerol kinase is the limiting step for growth of wildtype *E. coli* cells on glycerol [[4]], we assumed that increasing the growth rate could lead to higher productivity since the working cell volume would be reached faster. However, we found that the growth rate of our L-Phe producer strains (carrying plasmid pF81) was only weakly affected by the presence and overexpression of an additional *glpK* or *glpK*^G232D^ gene; the formation of L-Phe was even impaired despite a slightly increased glycerol consumption rate (Table 2). Thus, additional chromosomal copies of *glpK* (both wildtype or feedback-resistant) do not seem to enhance growth rate and thus may not be limiting for glycerol dissimilation, at least in our model strain, FUS4/pF81.

Furthermore, we could show that increasing the oxygen availability (from 20 to 60%) led to an increased glycerol consumption and - to a minor extent – also to an increased growth rate, but L-Phe productivity was lower (see Figure 2a,b, and Table 2). Under conditions of high pO_2_, the genes encoding enzymes for the TCA cycle and the respiratory chain are known to be expressed to a higher degree than under conditions with low oxygen availability [[65],[66]]. An increased flux through pyruvate, the TCA cycle and the aerobic respiratory chain, could explain the higher glycerol consumption. However, this would also lead to a drain of PEP which then would be no longer available for aromatic amino acid biosynthesis. This in turn would explain the lower L-Phe production. However, as we found mainly lactate and (to a smaller amount) acetate under high pO_2_ conditions, the TCA cycle/respiratory chain is unlikely to be the competitor as there is apparently enough of pyruvate to be shunted to lactate and acetate formation. Rather, an imbalance in the cellular metabolism has to be taken into account. Appearance of lactate under aerobic conditions could reflect an excess of reducing equivalents in the cell which cannot be disposed of by oxidative respiration [[50],[67]]. As glycerol is in a more reduced state than glucose, a surplus of electrons stemming from glycerol metabolism has to be handled. These reducing equivalents can be funnelled into the electron transport chain (ETC, with oxygen as final acceptor), e.g. via the membrane-bound G3P dehydrogenase GlpD [[5]]. As reducing equivalents in form of NADH are also gained through action of Ga3P dehydrogenase, reoxidation through the ETC. might get rate-limiting in the cell, and alternative endogenous electron acceptors as pyruvate could be used (to deliver lactate and NAD^+^). In conclusion, L-Phe production from glycerol under high oxygen conditions is impaired whereas growth rate is hardly affected.

*E. coli* cells growing on glycerol have to divert a portion of this carbon source to gluconeogenesis and to the PPP; this flux has been discussed to be insufficient [[18]]. While the aldolase reaction on DHAP and Ga3P is reversible and forms F1,6BP, an irreversible phosphatase reaction is necessary to form F6P, the precursor for glucose 6-phosphate or PPP metabolites [[19]–[21]]. In *E. coli* there are two enzymes that convert F1,6BP to F6P: FBPase I (Fbp, encoded by *fbp* gene) [[19],[20]] and FBPase II (gene *glpX*) [[21]]. Fbp is tightly regulated by opposing effectors. Known activators are 3-phosphoglycerate, PEP, citrate, cis-aconitate, isocitrate, oxaloacetate, and α-ketoglutarate [[68],[69]] whereas G6P and AMP are inhibitors with K_i_-values of about 40 μM and 3–8 μM, respectively [[69]]. On the other side, GlpX is inhibited by ADP and to a lesser extent by fructose-1-phosphate and ATP. While AMP has no influence, PEP activates the enzyme [[21]].

The doubled gene copy number and overexpression of *glpX* alone had only slightly positive effects on L-Phe production. While the growth rate increased only to a small extent, L-Phe productivity was increased by 10-20%. The positive effect of an extra chromosomal copy of *glpX* on growth and L-Phe production supports the idea that the flux to the PPP is one limiting step which can be overcome by a second gene copy. Marr [[18]] discussed the G6P supply to be limiting when *E. coli* is growing on glycerol. Our results point to a limiting supply of PPP intermediates such as E4P, especially for the production of aromatic amino acids as L-Phe. For *E. coli* growing on glucose as C source, the positive effect of extra copies (either chromosomal or plasmid-borne) of *tktA* on production of DAHP, shikimic acid, or L-Phe are well documented [[24]–[26],[64]]. STY of 3-deoxy-D-arabinoheptulosonate 7-phosphate (DAHP) on glycerol as sole C source was slightly enhanced (2.75 mmolC DAHP g_DCW_^−1^ h^−1^) over glucose (2.44 mmolC DAHP g_DCW_^−1^ h^−1^) when a glucose-positive evolvant of a Pts-deficient *E. coli* mutant (strain PB12 *aroB*-/pRW300aroG^fbr^) with a plasmid-borne *tktA* gene was used [[58]]. The total yield of DAHP (Y mmol C/DAHP mmolC C source), however, was less (0.25 compared to 0.52). Likewise, formation of 3-dehydroshikimic acid (DHS) and shikimate in the corresponding *aroB*^+^-strain was increased when grown on glycerol in comparison to glucose. However, these authors did not present data without increased *tktA* gene copy to allow a comparison with the wild type state [[58]].

We thus also examined whether an additional chromosomal copy of *tktA* would be beneficial to further increase E4P availability and therefrom L-Phe formation when *E. coli* cells grow on glycerol. The extra copy of *tktA* alone also resulted in slight improvements of carbon recovery and STY of L-Phe from glycerol. Again, the growth rate was slightly increased which we take as evidence that a limitation of the carbon flux towards the PPP exists when cells grow with glycerol as C source. Interestingly, the combination of both additional genes (*glpX*, *tktA*) gave a combined improvement in L-Phe productivity while the growth rate of this strain, FUS4.7/pF81, was lowest in comparison to the other strains. STY was highest, reaching 0.37 ± 0.01 g l^−1^ h^−1^, which is an increase of about 80% over the parent strain, FUS4/pF81. Thus the two genes, *glpX* and *tktA*, have a combined positive effect on product formation, presumably as the supply of both precursors of L-Phe, PEP and E4P, is more balanced. With regard to L-Phe productivity, the extra copy of *glpK* gene is of low impact, whereas the improved carbon flux towards the PPP appears to be more important.

Altering the C source (here substituting glucose by glycerol) of a producer strain should take into account that the cells have to readjust their pathways of anabolism (predominantly biomass formation) and catabolism (predominantly energy production). In the case of *E. coli* glycerol metabolism, the steps leading to the PPP from glycerol appear to be suboptimal as single gene additions (*glpX*, *tktA*) improve the growth rate on glycerol. Interestingly, *glpX* is the distal gene in the *glpFKX* operon [[54]]. Although this should lead to the concomitant induction of GlpX activity with the induction of glycerol facilitator and glycerol kinase activities, the total cellular fructose-1,6-bisphosphatase activity seems to be suboptimal with regard to growth rate on glycerol as carbon source.

The results shown here may contribute to a better understanding of *E. coli* product formation on the alternative C source, glycerol. We like to emphasize that the chromosomal alterations shown herein are thought to avoid metabolic burden for the cells as we only increase the gene copy number by one. Further incremental increases are possible when desired. Our strains thus can be further modified without having to re-engineer plasmids every time that a new positive effect of a given gene is found. Work is underway to find further limiting steps in the utilization of glycerol as alternative carbon source for *E. coli* [[31],[43]].

## Conclusions

Glycerol is a valuable carbon source that is readily taken for the production of the aromatic amino acid, L-Phe, by *E. coli* and crude glycerol from biodiesel plants can likewise be used. Extra copies of *glpK* (glycerol kinase) or *glpK*^G232D^ (encoding a fructose bisphosphate-resistant enzyme) did not improve productivity for L-Phe.

L-Phe productivity is however enhanced significantly by increasing gene copy number of *glpX* (encoding a gluconeogenic fructosebisphosphatase) or *tktA* (encoding transketolase from the PPP). Both genes were chromosomally integrated. Growth rates on glycerol, however, were not significantly enhanced. A combination of *glpX* and *tktA* gave best L-Phe productivities.

## Materials and methods

### Chemicals, enzymes, molecular biology

F1,6BP and Antifoam (polypropylene glycol P 2000) were from Sigma-Aldrich Chemie GmbH (Munich, Germany), IPTG was from PEQLAB Biotechnologie GmbH (Erlangen, Germany), Bradford solution was from Biorad and sodium dodecyl sulfate from SERVA Electrophoresis GmbH (Heidelberg, Germany). All other chemical were either from Carl Roth GmbH (Karlsruhe, Germany), Fluka (Taufkirchen, Germany), or Merck KGaA (Darmstadt, Germany) and were of the highest available purity. The crude glycerol was delivered from a biodiesel producer (ADM Hamburg, Germany) and contained 83.20% glycerol, 10.4% water, 6.4% NaCl, with less than 0.01% methanol, and no detectable traces of other organic compounds (according to supplier’s data sheet). All restriction enzymes used were from New England Biolabs (Frankfurt, Germany). The Pwo DNA Polymerase for gene amplification was from Roche Applied Science (Mannheim, Germany), Taq DNA polymerase used for screening-PCR was from Genaxxon Bioscience GmbH (Ulm, Germany).

### Strains and plasmids

All *E. coli* strains and plasmids used in this study are listed and described in Table 1, oligonucleotides are presented in Table 4.

**Table 4:** Oligonucleotides used in this study

**Name**	**Sequence**
*malE*-integr	5′-*AAGGTAAACTGGTAATCTGGATTAACGGCGATAAAGGCT ATAACGGTCTCGCTG*TCAAGGCGCACTCCCGTTCTGG-3′
*malG*-integr	5′-*GATCGGTAATGCAGACATCACGGCAGCGGCGGCAAAGTC ACCCCACAGGTAGTTTT*CAGGGTTATTGTCTCATGAGCG-3′
*rbsD*-integr	5′-*ACCGTTCTTAATTCTGATATTTCATCGGTGATCTCCCGTCT GGGACATACCGATA*TCAAGGCGCACTCCCGTTCTGG-3′
*rbsK*-integr	5′-*ATTCACGCTAGCCCATACACCACGACTTCCTAAAGTAATC AGTACAGTACGGATACC*CAGGGTTATTGTCTCATGAGCG-3′
*malK*-screen	5′-GCTTTCGTTACATTTTGCAGCTGTACG-3′
*cat*-control	5′-CCGTCACAGGTAGGCGCGCC-3′
*glpX*-for (NdeI)	5′-TTTT**CATATG**AGACGAGAACTTGCCATCGAATTTTCC-3′
*glpX*-rev (HindIII)	5′-TTTT**AAGCTT**CAATCAGAGGATGTGCACCTGCATTTCC-3′
*glpK*_for (NdeI)	5′-GCTGT**CATATG**ACTACGGGACAATTAAACATG-3′
*glpK*_rev (HindIII)	5′-CCGGATGCGGCATAA**AAGCTT**CATTCGGC-3′
*glpK*G232D-for	5′-GTATACGGTCAGACTAATATTGGCGACAAAGGCGGCAC-3′
*glpK*G232D-rev	5′-GTGCCGCCTTTGTCGCCAATATTAGTCTGACCGTATAC-3′

Sequence homologous to chromosomal integration location is shown in italics, bold letters indicate engineered restriction sites.

Standard molecular biology methods were used [[70]]. The vectors used for cloning were based on pJF119EH [[47]]. The PCR amplified genes were inserted in EcoRI/HindIII sites. To generate the protein variant (mutein) GlpK^G232D^ [[48]], the plasmid pKUS3 was mutagenized using the QuikChange-Site-Directed Mutagenesis Kit (Agilent Technologies, Germany) with the primer pair *glpK*G232D-for and *glpK*G232D-rev (Table 4). The DpnI-treated PCR product was transformed into XL1Blue cells (Stratagene, La Jolla, USA) (Table 1). Plasmid DNA of several clones was isolated with the NucleoSpin Kit (Macherey + Nagel, Düren, Germany). The correct nucleotide sequences of plasmid clones were verified by custom sequencing (GATC Biotech, Konstanz, Germany).

Recombineering methods were used for chromosomal disruption [[45]]. Site-specific integration of genes into the chromosome (knock-in by knock-out) of strain BW25113 have been described previously [[51]]. Knock-in mutants were selected on LB agar plates with chloramphenicol (Cm; 25 μg ml^−1^). Cm-resistant colonies were tested for correct location by PCR with appropriate primers (Table 4). Chromosomal modifications were first conducted in derivatives of strain BW25113 and then transferred by P1-mediated transductions into the FUS4 background [[52],[53]]. Strain constructs are listed and described in Table 1.

### Growth media

For L-Phe production, strains were grown aerobically (shake flasks cultures) in minimal medium (MM) at 37°C. MM consisted of: 3 g l^−1^ KH_2_PO_4_, 12 g l^−1^ K_2_HPO_4_, 5 g l^−1^ (NH_4_)_2_SO_4_, 0.3 g l^−1^ MgSO_4_*7H_2_O, 0.015 g l^−1^ CaCl_2_*2H_2_O, 0.1 g l^−1^ NaCl, 0,1125 g l^−1^ FeSO_4_*7H_2_O/sodium citrate, 0.0075 g l^−1^ thiamine, 5 g l^−1^ glucose (the concentration of glycerol was adjusted according to mmolC l^−1^ with glucose), 0.02 g l^−1^ L-tyrosine, 0.02 g l^−1^ L-Phe (modified after [[71]]). The pH was adjusted to 7.0 by the addition of H_3_PO_4_. LB (Luria Bertani)-medium consisted of (per liter) 10 g tryptone, 5 g yeast extract, and 10 g NaCl [[70]]. For maintenance of plasmid pF81, the medium was supplemented with ampicillin. Antibiotics were used at the following final concentrations: ampicillin 100 μg ml^−1^, kanamycin 50 μg ml^−1^, and chloramphenicol 25 μg ml^−1^.

### Preparation of cell-free extracts and enzyme assays

#### Fructose bisphosphatase (FBPase) activity

DH5α cells were transformed with the plasmid pKUS13, as a negative control vector pJF119EH was used. A single colony each was picked and inoculated in 5 ml LB medium with ampicillin. After overnight incubation on a rotary shaker at 37°C, 500 μl of the cultures were inoculated in 25 ml LB-medium with ampicillin and incubated on a rotary shaker at 37°C. After reaching OD_600_ = 0.5, enzyme formation was induced by addition of 0.5 mM IPTG (final concentration) and cells were further grown for 3 hours at 37°C. After harvesting the bacteria by centrifugation (10 min, 5000 rpm at 4°C), cells were resuspended in 2 ml TEA (triethanolamine)-buffer (82 mM, pH 7.6). Cells were disrupted by French press treatment, followed by ultrasonic treatment to disrupt chromosomal DNA (30 sec, 1 cycle, Bandelin Sonopuls HD 200, 200 W, 20 kHz (BANDELIN electronic GmbH & Co., Berlin, Germany) with constant cooling on ice. Cell debris was removed by centrifugation (30 min, 14000 rpm, 4°C) and the resulting cell-free extracts were immediately used. To measure FBPase activity from strains with chromosomally integrated *glpX*, cells were grown for 6 hours in 250 ml shaking flasks (filled with 25 ml LB) and IPTG was added from the start at a concentration of 0.5 mM. Cell disruption occurred as described above. Protein concentration was measured using the Bradford method [[72]]. The enzymatic activity was measured following the increase in phosphate concentration liberated from the substrate, F1,6BP [[55],[73]]. Assay conditions were as follows: 20 mM Tricine (pH 7.7), 50 mM KCl, 1 mM MnCl_2_, 1.5 mM F1,6BP, 100 μg of protein (present in the cell-free extract). Incubation was for 20 minutes at room temperature, aliquots of 50 μl were taken every 5 minutes and mixed with 800 μl of a solution consisting of 3 parts of a 0.045% malachite green solution and one part of a 4.2% ammonium molybdate in 4 N HCl. After 20 minutes the reaction was stopped by addition of 100 μl of sodium citrate solution (34%). The absorbance was read at 660 nm in a photometer [[55],[73]]. One unit (U) of specific enzyme activity is defined as the amount of enzyme required to release 1 μmol phosphate per minute per milligram of protein. Each measurement was performed in duplicate.

#### Glycerol kinase

Activity was measured in the crude extract. The various constructs were expressed in DH5α cells carrying the respective expression plasmids (wildtype variant, pKUS3 or the mutant variant, pKUS3-1). A single colony each was picked and inoculated in 5 ml LB-medium with ampicillin. After overnight incubation on a rotary shaker at 37°C 500 μl of the cell suspension was inoculated in 25 ml LB-medium with ampicillin and incubated on a rotary shaker at 37°C. After reaching OD_600_ = 0.5, 0.5 mM IPTG (final concentration) was added for induction, and cells were grown for 3 hours at 37°C. Cell disruption was done as described above. Protein concentration was measured using the Bradford method [[72]]. Glycerol kinase activity was measured using a glycerol assay kit (R-biopharm, Darmstadt, Germany), but using our crude extract instead of the standard kinase. The principle of the test was the following: Glycerol and ATP is converted by glycerol kinase to G3P and ADP followed by the conversion of ADP and PEP to ATP and pyruvate. In a last step L-lactate dehydrogenase converted Pyruvate and NADH to L-lactate and NAD^+^. The decrease in NADH was followed spectrophotometrically at 340 nm. The test was performed in duplicate following the manual instructions except that incubation was at 30°C to ensure constant conditions for all reactions. When F1,6BP was added as inhibitor of glycerol kinase, the reaction mixture was incubated for 5 minutes to ensure that the inhibitor could bind to its target site.

Overproduction of the appropriate gene products was checked by SDS gel electrophoresis [[74]].

### Batch and fed-batch growth experiments

Prior to use, the recipient strains (stored as glycerol stocks at −80°C) were streaked onto LB-agar plates and incubated overnight at 37°C. The cells were then transformed with plasmid pF81 and selected on LB agar plates supplemented with ampicillin. A single colony was picked and adapted to growth on MM on MM agar plates for 3 days at 30°C. For batch growth experiments in shaking flasks the preculture was grown in MM at 37°C in a volume of 5 ml. 25 ml MM (0.5% glycerol) in a 250 ml shaking flask was inoculated with the overnight culture to a starting OD_600_ = 0.1. When OD_600_ = 0.5 was reached IPTG was added (final concentration of 0.25 mM).

For fed-batch growth experiments, the preculture was grown at 37°C in a volume of 100 ml in a 1 l shaking flask using the same medium as for the fermentation. A benchtop bioreactor system (Labfors®, Infors AG, Suisse), with 1.5 l working volume and a total volume of 3.6 l, was used for all fed-batch experiments. Working conditions were: pH 7.0 (controlled by addition of 5 N KOH, 5 N H_3_PO_4_), 37°C, 60% oxygen saturation at ambient pressure (starting with pO_2_ = 100% at 200 rpm, when reaching 60% automatically controlled by stirring rate). Cultures were initiated from an exponential phase preculture at an OD_600_ of 0.05. After OD_600_ = 2.5 had been reached, IPTG was added to 0.1 mM final concentration. At OD_600_ = 10 the concentration of IPTG was augmented to a final concentration of 0.3 mM. L-Tyrosine was used for the control of biomass formation and was added to the medium until OD_600_ = 14–16 was reached (stock solution: 4 mg ml^−1^, sterile filtered). Initial glycerol concentration was 5 g l^−1^ and the glycerol concentration was thereafter kept between 3–6 g l^−1^ (stock solution: 50% (w/v), autoclaved). When necessary, ammonium sulphate (stock solution: 200 g l^−1^, autoclaved) was added as nitrogen source. Antifoam (polypropylene glycol P 2000, autoclaved) was added as required. All fermentations were performed two times independently. The space-time-yield (STY) was calculated for time periods of at least 10 hours.

### Analytical methods

Cell growth during the cultivations was monitored photometrically by following changes in OD_600_ of appropriate dilutions in MM (Cary 50 Bio, Varian). Glycerol concentration was measured using an enzymatic kit (R-biopharm, Darmstadt Germany). Lactate and acetate concentration was determined by HPLC analyses, performed on a Waters HPLC instrument (Milford, USA). The column was a Resex ROA organic acid, 300 × 7.8 mm, 8 μm, with a precolumn made of the same material (Phenomenex, Aschaffenburg, Germany). 5 mM H_2_SO_4_ was used as mobile phase with a flow rate of 0.5 ml min^−1^. Absorption was detected at 210 nm. For data analysis, the software Millenium32 Version 3.05.01 was used. L-Phe and L-tyrosine were determined with an HPLC system from Dionex (Germering, Germany) equipped with Chromeleon Software, Gina autosampler, P580 pumps, and a diode array detector. The column was a RP18 Lichrospher100, 250 × 4.6 mm, 5 μm (Merck, Darmstadt, Germany) with a precolumn of the same material. A flow rate of 1 ml min^−1^ was used. The mobile phase consisted of solvent A (water containing 0.1% (v/v) trifluoroacetic acid) and solvent B (acetonitrile containing 0.1% (v/v) trifluoroacetic acid). A total of 20 μl diluted sample was analyzed under gradient conditions. Equilibration conditions: 10 min 100% solvent A. 0–5 min 100% solvent A/0% solvent B, 5–30 min linear gradient to 70% solvent A/30% solvent B, 30–39 min linear gradient to 0% solvent A/100% solvent B, 39–46 min 0% solvent A/100% solvent B.

## Abbreviations

AMP: Adenosine monophosphate

ADP: Adenosine diphosphate

ATP: Adenosine triphosphate

Cm: Chloramphenicol

DAHP: 3-Deoxy-D-arabinoheptulosonate 7-phosphate

DHAP: Dihydroxyacetone phosphate

DHS: 3-Dehydroshikimic acid

EIIA^Glc^: Enzyme IIA^Glc^

E4P: Erythrose-4-phosphate

ETC: Electron transport chain

F1,6BP: Fructose-1,6-bisphosphate

F6P: Fructose-6-phosphate

FBPase: Fructose-1,6-bisphosphatase

G3P: Glycerol-3-phosphate

G6P: Glucose-6-phosphate

Ga3P: Glyceraldehyde-3-phosphate

Glc: Glucose

Gly: Glycerol

IPTG: Isopropyl β-D-1-thiogalactopyranoside

L-Phe: L-phenylalanine

MM: Minimal medium

NADH: Nicotinamide adenine dinucleotide

NADP: Nicotinamide adenine dinucleotide phosphate

Ni-NTA: Nitrilotriacetic acid

PEP: Phosphoenolpyruvate

PPP: Pentose phosphate pathway

PTS: Phosphotransferase system

SDS: Sodium dodecyl sulfate

STY: Space-time-yield

TCA: Tricarboxylic acid cycle

UQ_8_: Ubiquinone-8

X5P: Xylulose-5-phosphate

## Competing interests

The authors declare that they have no competing interests.

## Authors’ contributions

KG, CA, and GAS planned the experiments. KG performed most of the experiments and did data analysis. KG and GAS wrote the manuscript. All authors have read the manuscript and agreed on its final form.

## Additional file

## Supplementary Material

Additional file 1: **Growth of strains BW25113, BW25113** Δ***tktA*, and BW25113** Δ***tktA gal::tktA-cat*on LB-medium + 0.25% SDS.** Click here for file
