# Supplementary figures and images for: Improvement of L-phenylalanine production from glycerol by recombinant Escherichia coli strains: The role of extra copies of glpK, glpX, and tktA genes
Source: Microb Cell Fact. 2014 Jul 11;13:96. doi: 10.1186/s12934-014-0096-1 (PMC4227036; doi:10.1186/s12934-014-0096-1)

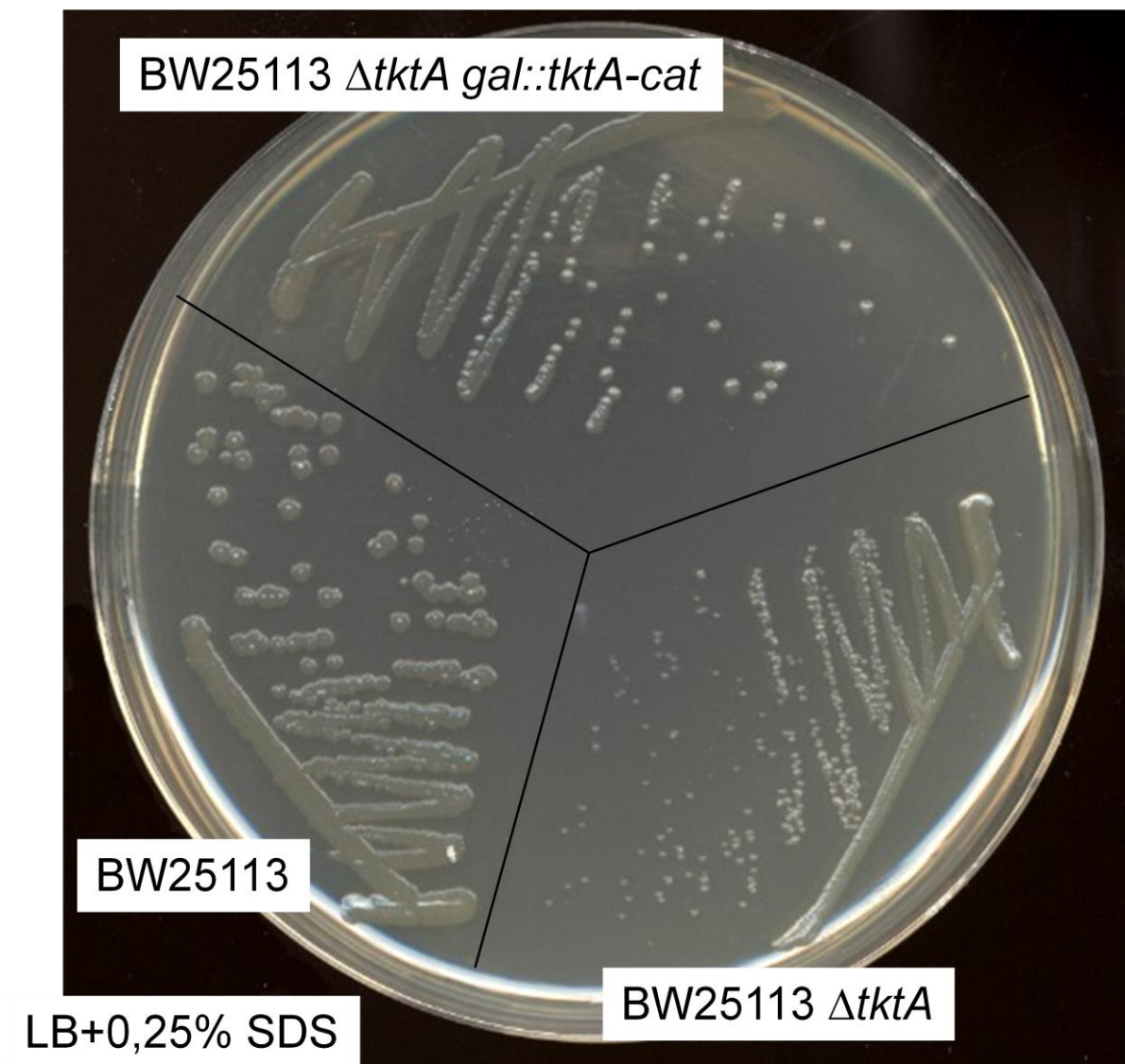

Supplement: Additional file 1: — Growth of strains BW25113, BW25113 ΔtktA, and BW25113 ΔtktA gal::tktA-caton LB-medium + 0.25% SDS. [file s12934-014-0096-1-S1.pdf]
